# Cross-sectional and longitudinal construct validity of two rotator cuff disease-specific outcome measures

**DOI:** 10.1186/1471-2474-7-26

**Published:** 2006-03-13

**Authors:** Helen Razmjou, Andrea Bean, Varda van Osnabrugge, Joy C MacDermid, Richard Holtby

**Affiliations:** 1 Orthopaedic and Arthritic Institute, Sunnybrook & Women's College Health Sciences Centre, Toronto, ON, M4Y 1H1, Canada; 2 Departments of Physical Therapy and Surgery, University of Toronto, Toronto, Canada; 3 School of Rehabilitation Science, McMaster University, Hamilton, Canada; 4 Hand and Upper Limb Centre, St. Joseph's Health Care, London, ON, N6A 4L6, Canada

## Abstract

**Background:**

Disease-specific Quality Of Life (QOL) measures are devised to assess the impact of a specific disease across a spectrum of important domains of life. The purpose of this study was to examine the cross-sectional and longitudinal construct validity (sensitivity to change) of two rotator cuff disease-specific measures, the Rotator Cuff-Quality Of Life (RC-QOL) and the Western Ontario Rotator Cuff (WORC) index, in relation to one another and to other joint and limb specific measures in the same population of the patients suffering from rotator cuff pathology.

**Methods:**

Participants enrolled were consecutive patients who received physical therapy for management of impingement syndrome or received treatment following rotator cuff repair, acromioplasty or decompression surgeries. All subjects received physical therapy treatment and completed four outcome measures at 3 single points (initial, interim, and final). Cross-sectional convergent validity was assessed at each of the 3 time-points by correlating the WORC and RC-QOL's scores to each other and to two alternative scales; a joint-specific scale, the American Shoulder and Elbow Surgeons (ASES) standardized shoulder assessment form and a limb-specific measure, the Upper Extremity Functional Index (UEFI). Non-parametric statistics (Spearman's rho and Wilcoxon-Mann-Whitney tests) examined the construct validity. The standardized response mean (SRM) was used to examine sensitivity to change.

**Results:**

Forty-one participants entered the study and their scores were compared at 3 cross sectional single points. The correlation coefficients among the 4 measures varied from 0.60 to 0.91. Correlation between corresponding domains of the WORC and RC-QOL varied from 0.45 to 0.85. The known group validity was not significantly different among individual sub-scores and total scores. The final SRMs were (1.42), (1.43), (1.44), and (1.54) for the ASES, RCQOL, WORC, and UEFI respectively.

**Conclusion:**

The WORC and RC-QOL exhibit similar cross-sectional convergent validity in patients suffering from rotator cuff pathology. The sensitivity to change was very close among all scores, with the UEFI having the highest sensitivity. Further research is needed to examine the extent to which each physical or emotional domain contributes to prognostic or therapeutic decision-making.

## Background

The assessment of health-related quality of life is becoming increasingly important in evaluating the effectiveness of orthopaedic interventions. Generic health-related quality of life (QOL) instruments such as the SF 36 have the ability to examine the extent of symptoms and disability among different diseases and conditions [1,2]. However, these measures have been shown to be less responsive than disease or joint-specific measures [3], particularly in the upper extremity area [4-6]. Limb and joint-specific QOL measures are often used in orthopaedics because they focus on the particular anatomical area [6,7]. A recently developed limb-specific self-report measure is the Upper Extremity Functional Index (UEFI) [8]. The most commonly used subjective joint-specific measure is the American Shoulder and Elbow Surgeons (ASES) standardized shoulder assessment form [9-11]. Disease-specific measures are available for rheumatoid arthritis [12], osteoarthritis [13,14], low back pain [15], neck conditions [16], carpal tunnel syndrome [17,18], and many other conditions. These measures have been reported to have excellent psychometric properties due to their ability to measure disease severity more accurately.

A number of conditions such as osteoarthritis, instability, and rotator cuff disease could affect the function of the shoulder joint and each condition has a distinct feature that characterizes a unique pathology. Consequently, there has been a growing interest in studies that examine specific aspects of quality of life affected by certain diseases of the shoulder joint [19-22]. As noted, the potential advantage of a disease specific measure is that it can address symptoms, impairments or activity limitations that are specific to the pathology of interest. However, the clinical value of this extra information in diagnosis or prognosis has not been investigated.

Rotator cuff disease, is the most common pathology in the shoulder joint [23] and leads to a significant disability affecting activities of daily living, work and sports, thereby influencing the quality of life [24]. Currently, there are two rotator cuff disease-specific outcome measures: the Western Ontario Rotator Cuff (WORC) Index [20] and the Rotator Cuff- Quality Of Life measure (RC-QOL) [19]. Although these questionnaires are designed to measure different domains of the QOL such as pain, physical, emotional, and social functioning, the original authors have not investigated the dimensionality and structure of the domains or items via factor analysis. In terms of validity, the WORC questionnaire was reported [20] to correlate most strongly with the American Shoulder and Elbow Surgeons, (ASES) and the Disability of Arm Shoulder and Hand (DASH). As an evaluative instrument it correlated best with the ASES and the University of California Los Angeles (UCLA). The correlations of the WORC's total score with the other instruments have been reported to range from 0.48 to 0.91 by other investigators [25,26]. Studies that have examined the responsiveness of the WORC by calculating the standardized response mean (SRM) in patients who have been measured before and after surgery have not reported noticeably different SRM from the comparative measures (Constant, SST and DASH) [25,26]. Holtby and Razmjou [25] had lower overall SRMs than MacDermid et al [26] who included only the responders in their calculations. Information on measurement properties of the RC-QOL is limited to the study conducted by the original authors [19]. This measure has demonstrated high test-retest reliability, face validity, and ability to discriminate between large and massive cuff tears as reported by the developers [19].

Although preliminary information on the validity of these measures has been reported, there are no independent studies investigating these properties in the same population. The purpose of this study was to examine the cross-sectional and longitudinal construct validity (sensitivity to change) of the WORC and RC-QOL in relation to one another and in relation to other joint and limb specific measures in the same population of the patients suffering from rotator cuff pathology.

## Methods

This prospective outcomes study (repeated-measures design) involved consecutive patients referred to the outpatient rehabilitation department of a tertiary care centre. These patients were referred by their family physicians or orthopedic surgeons for treatment of impingement syndrome or post-operative rehabilitation following rotator cuff-related surgeries. All patients completed the ASES, WORC, RC-QOL, and UEFI outcome measures at the initial and final visits. All measures except UEFI were collected at the interim visit. Patients with upper extremity fractures or systematic inflammatory disease such as rheumatic arthritis were excluded from the study.

The study protocol was approved by the Human Ethics Research Board of the Sunnybrook & Women's College Health Sciences Centre, Toronto, Canada.

### Description of the quality of life questionnaires

The Western Ontario Rotator Cuff (WORC) Index consists of 21 items representing five domains each with a visual analogue scale type response option [20]. The 5 domains include: 1) physical symptoms, 2) sports and recreation, 3) work, 4) social function, and 5) emotions. The WORC items are scored on a 100-point scale (0–100). The most symptomatic score is 2100 and the best or asymptomatic score is 0. In order to present this in a more clinically meaningful format, the score can be reported as a percentage of normal by subtracting the total score from 2100, dividing by 2100 and multiplying by 100. The final WORC scores can therefore vary from 0%, the lowest functional status level, to 100%, the highest functional status level. The RC-QOL was developed at the University of Calgary Sport Medicine Centre [19]. This measure consists of 34 items, representing five domains; 1) symptoms and physical complaints, 2) recreational activities, sports participation or competition, 3) work-related concerns, 4) lifestyle issues, and 5) social and emotional issues. Each item of the RC-QOL is scored with a 100-point visual analogue scale. The sum of all 34 items is divided by 34 to produce a total score out of 100. Questions that are not applicable do not need to be answered and will not be taken into account in the corresponding domains. The total final RC-QOL scores can therefore vary from 0, the highest functional status level, to 100, the lowest functional status level. The Upper Extremity Functional Index (UEFI) consists of 20 questions. Total score can vary from 0, the lowest functional status level, to 80, the highest functional status categorizing activities from "no difficulty" to "extreme difficulty" [8]. The American Shoulder and Elbow Surgeons (ASES) score was developed by the American Shoulder and Elbow Surgeons Committee for use in all types of shoulder problems [9]. The ASES is a 100-point standardized self-report form, 50 points of which are derived from patient self-report of pain on a visual analog scale and 50 points of which are computed from a formula using the cumulative score of 10 activities of daily living derived using a four-point ordinal scale. The higher scores of the ASES reflect less pain and better function. The superiority of the UEFI and the ASES is their practicality of being administered in and scored under 5 minutes as compared to 10 to 15 minutes for more lengthy measures.

## Results

### Analysis

Convergent validity examines the extent to which the outcome of interest agrees with the result of another measure that is believed to be assessing the same attribute. The cross-sectional convergent validity was evaluated by investigating the WORC and RC-QOL's ability to correlate with one another and other commonly used subjective measures, the ASES and the UEFI at a "single point in time"; initial, interim and final visits. It was hypothesized that the disease specific measures would have a higher correlation with the joint-specific measure, ASES than with the UEFI, which examines the entire upper extremity. In addition, it was hypothesized that the total scores of the WORC and RC-QOL would correlate closely with one another and that the relevant domains of the WORC and RC-QOL would have a marked (>0.60) or high degree (>0.80) of correlation according to Hinkel et al's [27]classification system. The following domains of the WORC were examined against the sections of the RC-QOL respectively: "physical symptoms" with "symptoms and physical complaints", "work" with "work-related concerns", "sports and recreation" with "recreational activities, sports participation or competition", "life style" with "life style issues", and "emotions" with "social and emotional issues". Association between total scores of all measures and the corresponding domains/sections of the WORC and RC-QOL were examined by non-parametric statistics (Spearman's rho Correlation Coefficients) in which normal distribution of data is not required. As a post-hoc analysis, we also evaluated the cross-sectional known group validity of the total scores of all measures and individual domains of the WORC and RC-QOL to examine the differences between the surgical and non-surgical groups. The Wilcoxon-Mann-Whitney test was used because the scores were not normally distributed.

As a measure of longitudinal construct validity (sensitivity to change), the standardized response mean (SRM) was calculated for all measures. The standardized response mean was calculated as the mean change scores divided by the standard deviation of the change scores. It was hypothesized that the WORC and RC-QOL would have a higher SRM due to their comprehensive nature and focus on impact of the disease.

Forty-one patients (range: 25 – 82, mean age = 57, SD = 16, 23 females and 18 males) were included in the study and 123 scores were obtained for the WORC and RC-QOL. Twelve patients received conservative treatment and 29 had post-operative rehabilitation. Out of 29 surgical patients, 18 patients had acromioplasty and 12 had rotator cuff repair. One patient had both surgeries. The affected side was the right in 26 patients and left in 12 patients. Three patients had bilateral problems in which the worst or the operated side was included in the study. There was a marked or high correlation among the total scores of all measures at a 0.01 level at all time points (Table 1). Both WORC and the RC-QOL had a higher correlation with the ASES than with the UEFI (Table 1). The correlations between the corresponding domains were considered marked or high at all time points except for initial physical symptoms, sports and recreation, and life style domains which showed a moderate correlation (Table 2). The SRM obtained for the UEFI was the highest (1.54) with the ASES being the lowest (1.42). The SRM was 1.43 and 1.44 for the RCQOL and WORC respectively (Table 3). There was no statistically significant (p = 0.056 to p = 0.94) difference among the domains of the WORC and RC-QOL and the total scores of all measures in differentiating between surgical and non-surgical groups. Table 4 demonstrates the domain of "emotions" in comparison with the total scores.

## Discussion

The exponential growth of quality of life studies over the last three decades appears to be the cause of increased interest in rigorous evaluation of therapeutic interventions. General-health questionnaires, such as the SF-36 provide accurate answers to the health-related questions posed. The disease-specific questionnaires provide similar concrete assessment for quality of life. The approach to the disease specific quality of life measures derives from the position that there are a number of domains of life that are affected by a specific disease process. Each domain contributes to one's overall assessment of the quality of life and consequently provides more accurate information. Recent evaluation of most shoulder disease or condition specific measures has failed to show a difference among "total scores" of the more lengthy measures and more generic ones [25,26,28]. However, breaking down the domains of some disease specific measures such as WORC has revealed statistically significant differences between different subgroups of patients [29]. In the present study, we examined the correlation between two self-report measures that are commonly used by Canadian physical therapists and surgeons who are involved in multicentre trials. The goal was to explore if the measures were consistent in terms of documenting the overall QOL and corresponding domains affected by the rotator cuff disease. Analysis of our first hypothesis confirmed a higher correlation between the shoulder joint measure (ASES) versus the UEFI as a limb specific measure (Table 1). The second hypothesis was also proved to be true except for 3 domains at initial assessment. The Spearman's rho between the domains of the physical symptoms, sports and recreation, and life style at the initial visit were considered lower than hypothesized (moderate at 0.45, 0.56, and 0.58 respectively). Interestingly, this increased to 0.79, 0.67, and 0.71 respectively at the final visit when the level of disability and pain reduced among the subjects. All other domains showed marked or high correlations at all time points. The explanation for this inconsistency may be in the nature of activities that are believed to cause symptoms. The RC-QOL encompass fairly strenuous activities such as mopping the floor, vacuuming the rug, scrubbing pots/pans, cleaning bathtub/toilet, carrying a heavy briefcase or small suitcase and raking the lawn or shovelling snow. These activities are expected to be significantly affected in patients with rotator cuff disease. In terms of sports and recreational activities, the advantage of the RC-QOL is its flexibility of providing the "not applicable" option to patients who do not perform a certain task. The instruction of the WORC questionnaire is to make the "best guess" on items that do not pertain to the patient. This however is troublesome for older individuals who have never performed a task such as push-ups. The number of patients who completed the sports section of the RC-QOL at initial assessment was 25 as compared to 41 who had to make a guess while completing the WORC and this could have contributed to a lower correlation between these two domains. The domains of "life style" are slightly different between the two measures as they address more routinely performed activities such as dressing and undressing, sleeping, and styling hair in the WORC and more physically demanding activities such as climbing a ladder and using power tools in the RC-QOL. Obviously, as patients improved in their ability to perform these tasks, the correlations improved.

Examining the known group validity did not prove that the individual domains of the WORC or RC-QOL were better in discriminating surgical and non-surgical patients with respect to documenting the impact of disease. A previous study [29] of 279 surgical candidates for rotator cuff showed that the domain of "emotions" of the WORC, which documents the psychological impact of disease could differentiate between different genders and age groups. The small sample size of the present study may be a contributing factor to our insignificant results and consequently, the role of domains warrants further evaluation in larger populations. Longitudinal construct validity was very closely matched across all 4 scales, with the UEFI having the highest sensitivity. Our third hypothesis was therefore not supported in that the disease-specific measures did not demonstrate greater sensitivity to change. However, the clinical importance of small differences (i.e, 0.10) needs to be further investigated.

High correlation of the disease-specific measures with much shorter self-report measures such as ASES and the UEFI may suggest a clinical advantage of the shorter measures as the respondent and clinician burden are lessened. It may be that because shoulder symptoms are such a predominant feature of the person's quality of life [18], that shorter instruments that focus on shoulder/arm pain and disability may capture the overall impact of the disease. Overlapping of pain and disability as perceived by the patients has been reported in different populations [30,31] and may affect the structure and dimensionality of the questions and domains of the measures that did not have factor analysis as a part of initial validity analysis. Future studies are needed to further examine the role of information that each domain of disease-specific measures provides to clinicians and investigators. Rigorous Rasch analysis of the WORC and RC-QOL may provide further insight into dimensionality and structure of the domains and produce a shorter version that is more suitable for busy clinicians.

## Conclusion

Based on the results of this study, the WORC and RC-QOL exhibit similar cross-sectional and longitudinal construct validity as compared with joint or limb measures. The sensitivity to change was very close among all scores, with the UEFI having the highest sensitivity.

In the present study, we demonstrated that the corresponding domains of the WORC and RC-QOL were concordant (moderate to high correlations). However, the domains of these two disease-specific measures did not demonstrate a higher known-group validity in discriminating between surgical and non-surgical groups. The main value of disease-specific QOL measures is in their ability to document the impact of disease on each domain of quality of life. If different domains are recognized as being informative in clinical research, information of subscales should be reported and analyzed. Therefore, the role of domains and the extent that each subscale scores contribute to prognostic and therapeutic decision-making warrants further evaluation.

## Competing interests

The author(s) declare that they have no competing interests.

## Authors' contributions

HR proposed the study, developed the research protocol, managed the database, analysed the data, and wrote the first draft of the paper. AB, VVO, and HR were involved in the grant proposal writing and patient recruitment. JCM provided input on analysis and content of the first draft. RH was involved in patient recruitment and providing surgical diagnosis. All authors were involved in the preparation of the manuscript and approved the final version.

## Pre-publication history

The pre-publication history for this paper can be accessed here:



## Figures and Tables

**Table 1:** Cross-sectional Spearman's rho correlation coefficients

		**WORC**	**RCQOL**	**UEFI**	**ASES**
**WORC:**					
**Initial**			.708(**)	.618(**)	.756(**)
	N		41	40	40
					
**Interim**			.872(**)		.738(**)
	N		41		41
					
**Final**			.913(**)	.779(**)	.813(**)
	N		40	39	41

**RCQOL:**					
**Initial**		.708(**)		.606(**)	.622(**)
	N	41		40	40
					
**Interim**		.872(**)			.822(**)
	N	41			41
					
**Final**		.913(**)		.672(**)	.697(**)
	N	40		38	40

** Correlation is significant at the 0.01 level (2-tailed).

**Table 2:** Spearman's rho correlations between domains of RC-QOL and WORC

		**WORC PS**	**WORC W**	**WORC S**	**WORC L**	WORC E*
**RCQOL (A)**						
**Initial**		-.450**				
	N	41				
						
**Final**		-.797**				
	N	40				
						
**RC-QOL (B)**						
**Initial**			-.727**			
	N		30			
						
**Final**			-.853**			
	N		30			
						
**RC-QOL (C)**						
**Initial**				-.562**		
	N			25		
						
**Final**				-.669**		
	N			25		
						
**RC-QOL (D)**						
**Initial**					-.582**	
	N				39	
						
**Final**					-.713**	
	N				40	
						
**RC-QOL (E)**						
**Initial**						-.669**
	N					40
						
**Final**						-.771**
	N					40

** Correlation Coefficient is significant at the 0.01 level (2-tailed).

A: Symptoms and physical complaints, PS: Physical symptoms, B: Work-related concerns, W: Work, C: Sports participation or competition, S: Sports and recreation, D: Life style issues, L: Lifestyle, E: Social and emotional issues, E*: Emotions

**Table 3:** Descriptive statistics related to mean change and standard deviation of change

	Mean Change	SD of Change	SRM
UEFI	30.526	19.801	1.54
WORC	28.487	19.739	1.44
RC-QOL	30.625	21.344	1.43
ASES	28.552	20.101	1.42

SD: Standard Deviation

SRM: Standardized Response Mean

**Table 4:** Cross-sectional known group validity between total scores and emotion domains of the WORC and RC-QOL (surgical and non-surgical groups)

**Test Statistics^b^**						
	WORC	RCQOL	UEFI	ASES	WORC E	RCQOLE

Mann-Whitney U	161.500	154.500	138.500	151.000	155.500	103.000
Wilcoxon W	239.500	232.500	573.500	229.000	590.500	181.000
Z	-.358	-.559	-1.018	-.502	-.530	-1.919
Asymp. Sig. (2-tailed)	.720	.576	.309	.615	.596	.055
Exact Sig. [2*(1-tailed Sig.)]	.724^a^	.581^a^	.314^a^	.631^a^	.601^a^	.056^a^

a. Not corrected for ties.

b. Grouping Variable: Surgery Group1

WORC E: Emotion domain of the WORC

RCQOL E: Emotion domain of the RCQOL

## References

[B1] Ware JE (2003). Conceptualization and measurement of health-related quality of life: comments on an evolving field. Arch Phys Med Rehabil.

[B2] Ware JE, Sherbourne CD (1992). The MOS 36-item short-form health survey (SF-36). I. Conceptual framework and item selection. Med Care.

[B3] Davies GM, Watson DJ, Bellamy N (1999). Comparison of the responsiveness and relative effect size of the western Ontario and McMaster Universities Osteoarthritis Index and the short-form Medical Outcomes Study Survey in a randomized, clinical trial of osteoarthritis patients. Arthritis Care & Research.

[B4] Amadio PC, Silverstein MD, Ilstrup DM, Schleck CD, Jensen LM (1997). Outcome after Colles fracture: the relative responsiveness of three questionnaires and physical examination measures. J Hand Surg Am.

[B5] Atroshi I, Johnsson R, Sprinchorn A (1998). Self-administered outcome instrument in carpal tunnel syndrome. Reliability, validity and responsiveness evaluated in 102 patients. Acta Orthop Scand.

[B6] Gay RE, Amadio PC, Johnson JC (2003). Comparative responsiveness of the disabilities of the arm, shoulder, and hand, the carpal tunnel questionnaire, and the SF-36 to clinical change after carpal tunnel release. J Hand Surg [Am].

[B7] MacDermid JC, Richards RS, Donner A, Bellamy N, Roth JH (2000). Responsiveness of the short form-36, disability of the arm, shoulder, and hand questionnaire, patient-rated wrist evaluation, and physical impairment measurements in evaluating recovery after a distal radius fracture. J Hand Surg [Am].

[B8] Stratford PW, Binkley JM, Stratford DM (2001). Development and initial validation of the upper extremity functional index. Physiotherapy Canada, Fall.

[B9] Richards RR, An K, Bigliani LU, Friedman R, Gartsman GM, Gristina AG, Iannotti JP, Mow VC, Sidles JA, Zuckerman JD (1994). A standardized method for the assessment of shoulder function. Journal of Shoulder Elbow Surgery.

[B10] Michener LA, Leggin BG (2001). A review of self-report scales for the assessment of functional limitation and disability of the shoulder. J Hand Ther.

[B11] Michener LA, McClure PW, Sennett BJ (2002). American Shoulder and Elbow Surgeons Standardized Shoulder Assessment Form, patient self-report section: reliability, validity, and responsiveness. J Shoulder Elbow Surg.

[B12] Salaffi F, Stancati A, Carotti M (2002). Responsiveness of health status measures and utility-based methods in patients with rheumatoid arthritis. Clin Rheumatol.

[B13] Bellamy N, Buchanan WW, Goldsmith CH, Campbell J, Stitt LW (1988). Validation study of WOMAC: a health status instrument for measuring clinically important patient relevant outcomes to antirheumatic drug therapy in patients with osteoarthritis of the hip or knee. J Rheumatol.

[B14] Beaton DE, Schemitsch E (2003). Measures of health-related quality of life and physical function. Clin Orthop Relat Res.

[B15] Beurskens AJ, de Vet HC, Koke AJ, van der Heijden GJ, Knipschild PG Measuring the functional status of patients with low back pain. Assessment of the quality of four disease-specific questionnaires. Spine.

[B16] BenDebba M, Heller J, Ducker TB, Eisinger JM Cervical spine outcomes questionnaire: its development and psychometric properties. Spine.

[B17] Katz JN, Gelberman RH, Wright EA, Lew RA, Liang MH (1994). Responsiveness of self-reported and objective measures of disease severity in carpal tunnel syndrome. Med Care.

[B18] MacDermid JC, Tottenham V (2004). Responsiveness of the Disability of the Arm, Shoulder, and Hand (DASH) and Patient-Rated Wrist/Hand Evaluation (PRWHE) in evaluating change after hand therapy. J Hand Ther.

[B19] Hollinshead RM, Mohtadi NG, Vande Guchte RA, Wadey VM (2000). Two 6-year follow-up studies of large and massive rotator cuff tears: comparison of outcome measures. J Shoulder Elbow Surg.

[B20] Kirkley A, Alvarez C, Griffin S (2003). The Development and Evaluation of a Disease-specific Quality-of-Life Questionnaire for Disorders of the Rotator Cuff: The Western Ontario Rotator Cuff Index. Clin J Sport Med.

[B21] Kirkley A, Griffin S, McLintock H, Ng L (1998). The development and evaluation of a disease-specific quality of life measurement tool for shoulder instability. The Western Ontario Shoulder Instability Index (WOSI). Am J Sports Med.

[B22] Lo IK, Griffin S, Kirkley A (2001). The development of a disease-specific quality of life measurement tool for osteoarthritis of the shoulder: The Western Ontario Osteoarthritis of the Shoulder (WOOS) index. Osteoarthritis Cartilage.

[B23] Zakaria D (2004). Rates of carpal tunnel syndrome, epicondylitis, and rotator cuff claims in Ontario workers during 1997. Chronic Dis Can.

[B24] MacDermid JC, Ramos J, Drosdowech D, Faber K, Patterson S (2004). The impact of rotator cuff pathology on isometric and isokinetic strength, function, and quality of life. J Shoulder Elbow Surg.

[B25] Holtby R, Razmjou H (2005). Measurement properties of the Western Ontario rotator cuff outcome measure: A preliminary report. J Shoulder Elbow Surg.

[B26] MacDermid JC, Drosdowech D, Faber K Responsiveness of self-report scales in patients recovering from rotator cuff surgery. J Shoulder Elbow Surg.

[B27] Hinkle, Wiersma, Jurs (1988). Applied Statistics for the Behavioral Sciences.

[B28] Bot SD, Terwee CB, van der Windt DA, Bouter LM, Dekker J, de Vet HC (2004). Clinimetric evaluation of shoulder disability questionnaires: a systematic review of the literature. Ann Rheum Dis.

[B29] Razmjou H, Holtby R, Myhr T (2006). Gender Differences in Functional Status and Extent of Pathology in Patients Undergoing Rotator Cuff Surgery. Arthroscopy.

[B30] Kennedy D, Stratford PW, Pagura SM, Wessel J, Gollish JD, Woodhouse LJ (2003). Exploring the factorial validity and clinical interpretability of the Western Ontario and McMaster Universities Osteoarthritis Index (WOMAC). Physiother Can.

[B31] Wessel J, Razmjou H, Mewa Y, Holtby R The factor validity of the Western Ontario Rotator Cuff Index. BMC Musculoskelet Disord.

